# Supplementary material for: Conditional chemoconnectomics (cCCTomics) as a strategy for efficient and conditional targeting of chemical transmission
Source: eLife. 2024 Apr 30;12:RP91927. doi: 10.7554/eLife.91927 (PMC11060718; doi:10.7554/eLife.91927)
Supplement: Figure 3—source data 3. [file elife-91927-fig3-data3.zip › Figure 3 source data3 part 1.zip!/Figure 3-Source data 3-part1/1082-9-CRISPResso_Report_zod2ewbs.zip!/CRISPResso_on_1082_11/1c.Alignment_barplot.pdf]

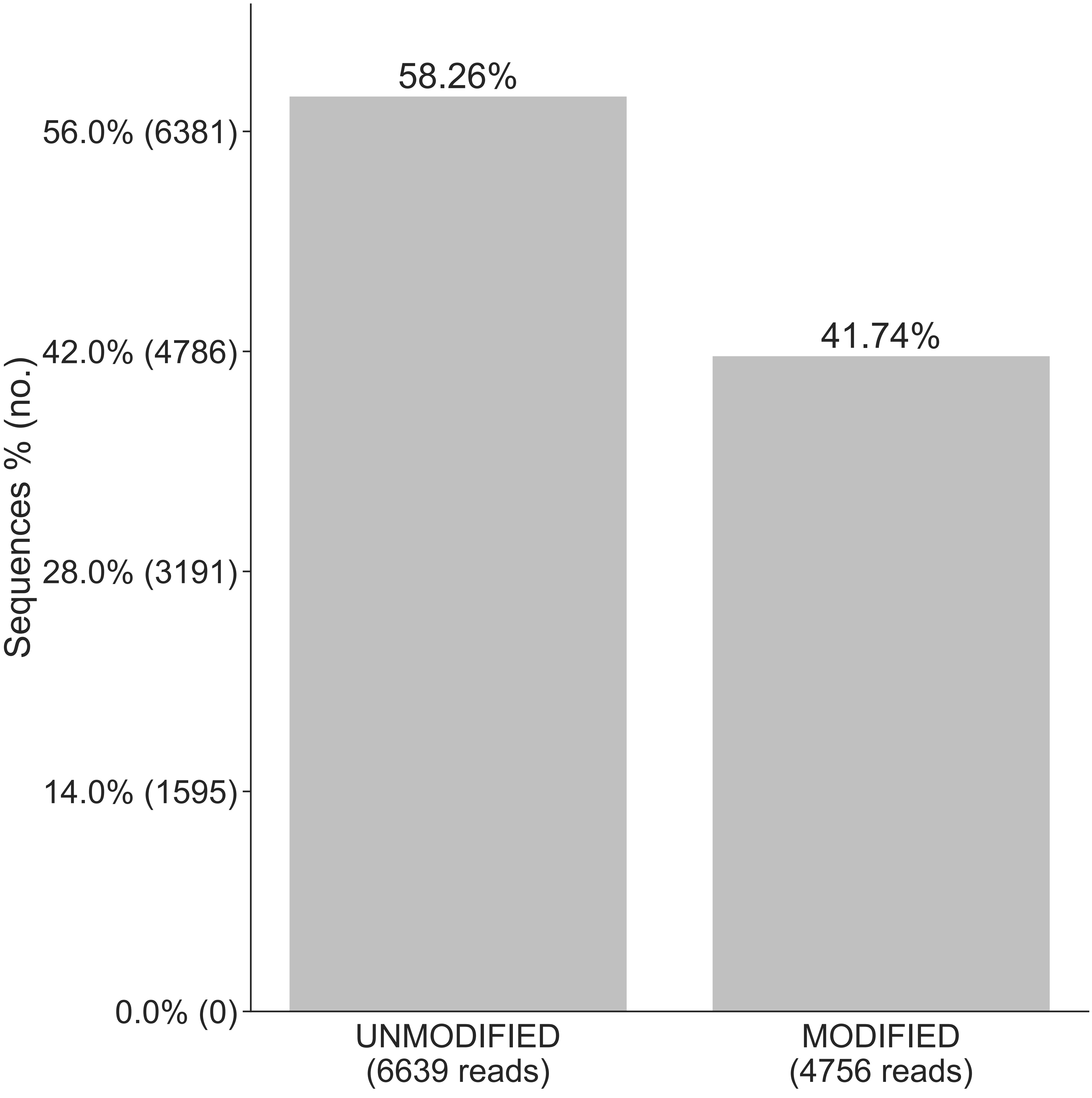
Sequences % (no.)
56.0% (6381)
42.0% (4786)
28.0% (3191)
14.0% (1595)
0.0% (0)
58.26%
41.74%
UNMODIFIED
(6639 reads)
MODIFIED
(4756 reads)
